# Senegalin-2: A Novel Hexadecapeptide from *Kassina senegalensis* with Antibacterial and Muscle Relaxant Activities, and Its Derivative Senegalin-2BK as a Bradykinin Antagonist

**DOI:** 10.3390/biom15010030

**Published:** 2024-12-30

**Authors:** Yueyang Lu, Yanguo Zhu, Chengbang Ma, Lei Wang, Mei Zhou, Tianbao Chen, Xiaonan Ma, Xu Zhang, Zhimin Fan

**Affiliations:** 1 Jiangsu Clinical Innovation Center for Anorectal Diseases of T.C.M., Nanjing Hospital of Chinese Medicine Affiliated to Nanjing University of Chinese Medicine, Nanjing 210022, China; ylu21@qub.ac.uk; 2 School of Pharmacy, Queen’s University Belfast, 97 Lisburn Road, Belfast BT9 7BL, UK; yzhu36@qub.ac.uk (Y.Z.); c.ma@qub.ac.uk (C.M.); l.wang@qub.ac.uk (L.W.); m.zhou@qub.ac.uk (M.Z.); t.chen@qub.ac.uk (T.C.); xiaonan.ma@qub.ac.uk (X.M.); 3 School of Medicine and Life Sciences, Nanjing University of Chinese Medicine, Nanjing 210023, China; zhangxu@njucm.edu.cn

**Keywords:** amphibian, antimicrobial peptides, smooth muscle, bradykinin, antagonist

## Abstract

The amphibian skin secretions are excellent sources of bioactive peptides, some of which and their derivatives exhibit multiple properties, including antibacterial and antagonism against bradykinin. A novel peptide Senegalin-2 was isolated from the skin secretions of *Kassina senegalensis* frog. Senegalin-2 relaxed rat bladder smooth muscle (EC_50_ 17.94 nM) and ileum smooth muscle (EC_50_ 135 nM), inhibited *S. aureus* and MRSA at 2 μM, and exhibited low hemolytic activity with no cytotoxicity. To design effective bradykinin antagonists, Senegalin-2 was conjugated with bradykinin to synthesize Senegalin-2BK. This modification retained potent activity against Gram-positive bacteria. Compared to Senegalin-2, Senegalin-2BK significantly reduced hemolysis and exhibited a more than threefold increase in the selectivity index. Furthermore, Senegalin-2BK contracted the bladder (EC_50_ 2.83 μM) and ileum (EC_50_ 56.64 nM)’s smooth muscle. The pretreatment with 10^−7^ M Senegalin-2BK reduced the 10^−6^ M bradykinin contraction on the bladder by over 70%. In conclusion, Senegalin-2 has dual functionalities as an antibacterial agent and muscle relaxant, positioning it as a potential therapeutic candidate for managing overactive bladder. As a synthetically derived bradykinin antagonist and myotropic peptide with antibacterial properties, Senegalin-2BK shows promise in effective therapies for relieving pain, inflammation, and addressing muscular disorders such as urinary retention, constipation, and infections.

## 1. Introduction

Bradykinin (BK), a crucial bioactive peptide within the human kallikrein–kinin system, exerts a dual-edged effect on the cardiovascular and nervous systems. Under physiological conditions, BK binds to its receptors to mediate vasodilation, improve myocardial energy metabolism, and enhance neural blood supply, thereby exerting protective effects [1,2]. Conversely, elevated BK levels in pathological states become a driving force for disease progression, exacerbating inflammation, acute infections, and pain [3]. This underscores the necessity of moderate BK receptor inhibition as an efficacious therapeutic strategy. Currently, several BK receptor antagonists, such as icatibant, have been clinically applied to swiftly and effectively manage acute attacks of hereditary angioedema (HAE). However, the limited number of clinically approved BK antagonists highlights the urgent need for the development of novel and potent BK antagonists to address unmet medical needs [4].

Amphibian skin secretions have been recognized as a rich source of bioactive peptides [5]. Among these bioactive peptides derived from amphibian skin secretions, a subset of peptides is involved in the regulation of smooth muscle tone, which is crucial for protecting amphibian species from predators. Since the 1960s, amphibian myotropic peptides have been discovered and classified into bradykinins (BKs), bradykinin-related peptides (BRPs), and other structurally unique amphibian myotropic peptides [6,7]. Amphibian BKs share the same sequence as mammalian BK, RPPGFSPFR. In contrast, amphibian BRPs contain a highly conserved BK sequence with additional N-terminal or C-terminal extensions. These peptides often exhibit amino acid additions, deletions, and substitutions, resulting in diverse primary structures and, consequently, a wide range of biological activities. For instance, the amphibian BRP ornithokinin (RPPGFTPLR) induced contraction of the ileum and uterine smooth muscle. However, another reported amphibian BRP, RVAL-(L1, T^6^, L^8^)-bradykinin, exhibited a slightly divergent sequence compared to ornithokinin, yet demonstrated markedly altered biological activity. RVAL-(L1, T^6^, L^8^)-bradykinin lacked intrinsic myotropic activity but effectively antagonized the vasorelaxant effect of bradykinin (BK) on arterial smooth muscle [8,9]. Notably, only a few natural amphibian BRPs, such as the aforementioned RVAL-(L1, T^6^, L^8^)-bradykinin, RAP-L1, T6-BK, exerted bradykinin antagonistic effects. These amphibian BRPs partially overlapped with BK in sequence and effectively inhibited BK-induced activities by competitively binding to the bradykinin B2 receptor (B2R). However, the antagonistic effects of these BRPs were typically observed at relatively high concentrations, and their physicochemical properties and toxicity remained unclear [10,11]. This presented challenges for designing and developing potent and low-toxicity BK antagonistic peptides.

Apart from BK and BRPs, researchers identified some unique myotropic peptides in amphibian skin. These peptides typically consist of 8–20 amino acids and have limited similarity to known peptides in the NCBI database. *Kassina* frog is a notable source of such myotropic peptides. Peptides such as Senegalins, Kassorins, and WL-8 have been isolated from *Kassina* frogs, inducing changes in rat bladder or arterial smooth muscle tension. Moreover, some of these peptides demonstrated multiple activities, including antimicrobial effects [12,13,14].

Many amphibian myotropic peptides have been reported to be involved in regulating bladder smooth muscle tone. Potassium channels are key molecules in modulating bladder smooth muscle function, controlling muscle tension by maintaining the cell’s resting membrane potential and shaping action potentials. Potassium channel blockers, such as aminopyridines, specifically inhibit the function of potassium channels, thereby affecting smooth muscle activity. If the effects of myotropic peptides are suppressed in smooth muscles pretreated with aminopyridines, it can be inferred that the myotropic effects of amphibian myotropic peptides rely on potassium channels.

In this study, we isolated a novel peptide, Senegalin-2, from the *Kassina senegalensis* frog. Pharmacological screening revealed its low cytotoxicity and dual activities: potent antibacterial efficacy against Gram-positive bacteria and the ability to induce bladder smooth muscle relaxation. To harness these beneficial properties while also creating a bradykinin (BK) antagonist, we designed a fusion peptide, Senegalin-2BK, by conjugating Senegalin-2 with BK. Antibacterial assays confirmed that Senegalin-2BK not only retains the strong antibacterial activity of Senegalin-2, but also broadens its antimicrobial spectrum. Furthermore, pharmacodynamics experiments on various smooth muscle tissues demonstrated that, unlike Senegalin-2, Senegalin-2BK exhibited contractile effects on both bladder and ileum smooth muscles. By observing the impact of aminopyridine incubation on smooth muscle tension assays, the pivotal role of potassium channels in the myotropic actions of these peptides was elucidated. Senegalin-2BK’s inhibition of BK-induced smooth muscle contraction validated its potential as a BK antagonist. Preliminary insights into the binding mode between Senegalin-2BK and the BK receptor, obtained through peptide structure simulation and receptor docking studies, lay the foundation for future research. Our objective is to develop multi-functional myopeptides that offer novel therapeutic strategies for BK-related diseases and contribute to the advancement of peptide-based therapies.

## 2. Materials and Methods

### 2.1. Acquisition of Frog Skin Secretions

Transcutaneous electrical stimulation (5 V DC; 140 ms pulse duration; 100 Hz) was applied to the dorsal skin of adult *Kassina senegalensis* frogs (*n* = 5, mixed male and female) every 20 s for 2 min. The frog’s back was then rinsed with deionized water to collect secretions and flash-frozen in liquid nitrogen. The above steps were carried out following the UK Animals (Scientific Procedures) Act 1986, Project Permit PPL 2694 issued by the Department of Health, Social Services, and Public Safety of Northern Ireland. The Institutional Animal Care and Use Committee (IACUC) of Queen’s University Belfast approved the procedures on 1 March 2011.

### 2.2. Construction of Kassina senegalensis Frog Skin cDNA Library and the “Shotgun” Cloning of Senegalin-2

The polyadenylated mRNA of the lyophilized *Kassina senegalensis* frog skin secretion was isolated. Then, the isolated mRNA was synthesized into cDNA by using the Dynabeads^®^ mRNA Direct^TM^ kit (Dynal Biotech, Merseyside, UK). The cDNA library was then established by rapid amplification of cDNA ends-PCR (RACE-PCR). A SMART-RACE kit (Clontech, Palo Alto, CA, USA) was used to obtain the complete peptide nucleic acid sequence. Nested universal primers (NUP) and sense primers (AC-S AGC AGC AAA AGA AGA AGA AGC CAT G) were applied in 3′-RACE reactions to obtain the target sequence. Afterward, the pGEM^®^-T Easy Vector System (Promega, Southampton, UK) was applied to purify and clone the RACE product. An ABI 3100 automated capillary sequencer (Applied Biosystems, Foster City, CA, USA) was then run to analyze the nucleotide sequence of Senegalin-2. 

Bioinformatics analysis of the putative peptide was conducted with the online tool Basic Local Alignment Search Tool (BLAST) and the sequence alignment software Clustal Omega (https://www.ebi.ac.uk/jdispatcher/msa/clustalo, accessed on 20 May 2024).

### 2.3. Isolation and Sequencing of Senegalin-2 from Kassina senegalensis Frog Skin Secretions

Similar to the previous study [10], 5 mg lyophilized frog skin secretions were dissolved in 0.05% *(v*/*v)* trifluoroacetic acid (TFA) and centrifuged. The supernatant obtained from centrifugation was pumped into a reverse-phase high-performance liquid chromatography (RP-HPLC) system (Amersham Biosciences, Buckinghamshire, UK) equipped with a C18 column, with fractions being automatically collected every minute. Subsequently, Matrix-Assisted Laser Desorption/Ionization Time-Of-Flight Mass Spectrometry (MALDI-TOF MS) (Perseptive Biosystems, Foster City, CA, USA) and a LCQ Fleet Ion Trap Mass Spectrometer (Thermo Fisher Scientific, San Francisco, CA, USA) were utilized for mass-to-charge ratio determination and MS/MS fragment sequencing of the fractions, respectively. Based on these results, combined with the Proteome Discoverer software 1.0 software, the molecular mass and sequence of Senegalin-2 were analyzed and identified.

### 2.4. Synthesis, Purification, and Validation of Peptides

The peptides were synthesized via Fmoc solid-phase peptide synthesis (SPPS) using a solid-phase synthesizer (Protein Technologies, Tucson, AZ, USA). Amino acids for the peptide chain were mixed with HBTU coupling reagent and rink amide MBHA resin to catalyze coupling and C-terminal amidation. Synthesis steps included DMF washing, 20% piperidine deprotection, 11% NMM dissolution, and activation. Peptides were ligated from C- to N-terminal, washed with degassed DCM, and cleaved using a cleavage cocktail (TFA/ddH_2_O/TIS/EDT, 94/2/2/2, *v*/*v*). The peptides were then washed, lyophilized, and stored at −20 °C.

Crude peptides were purified by the RP-HPLC system to obtain peptides with 95% purity (Agilent Technologies, Deutschland, Waldbronn, Germany). The molecular mass of the peptides was verified by the Mass Spectrometer (Waters, Milford, MA, USA).

### 2.5. Structural Analysis and Detection of Peptides

The physicochemical properties of peptides were predicted by Heliquest (available online: https://heliquest.ipmc.cnrs.fr/cgi-bin/ComputParams.py, accessed on 14 May 2024) [15]. As previously described, the secondary structure of peptides was measured: The peptides were, respectively, dissolved in 10 mM NH_4_Ac and TFE/10 mM NH_4_Ac (50/50, *v*/*v*) solution. Subsequently, the peptides were analyzed using a circular dichroism (CD) spectrometer (JASCO Inc., Easton, MD, USA), scanning wavelengths from 190 to 250 nm. The sample values were then obtained cumulatively through three scans. Finally, the helix ratios for these peptides were determined by BestSel (accessed on 25 May 2024, https://bestsel.elte.hu/index.php) [16].

### 2.6. Hemolysis Assays of Peptides

As mentioned before [17], horse erythrocytes were extracted from fresh horse blood, washed repeatedly with PBS via centrifugation at 1000× *g* until supernatant clarity, and resuspended to create a 4% (*v*/*v*) erythrocyte suspension. Peptides were diluted with PBS to form solutions and serially diluted. Erythrocyte suspension and peptide or control solutions were mixed, incubated at 37 °C for 2 h, centrifuged at 900× *g*, and absorbance at 550 nm was measured using a microplate spectrophotometer.

### 2.7. Cytotoxicity Assays of Peptides

Human microvascular endothelial cell line HMEC-1 (ATCC-CRL-3243) was cultured in MCDB 131 medium and seeded in a 96-well plate at 8000 cells per well for overnight incubation. Subsequently, peptides at final concentrations of 100, 10, 5, and 1 μM were added to the plate. The positive control was 0.1% (*w*/*v*) Triton-X-100. The negative control was the PBS solution. Growth control was the equal amount of medium. After 24 h of incubation, 10 μL of MTT solution (5 mg/mL) was added to each well and incubated in the dark for 2 h. The solution was then removed, and 100 μL of DMSO was added. After shaking the plate for 10 min, the optical density (OD) at λ = 570 nm was measured using a Synergy^HT^ microplate reader (Biotech, Minneapolis, MN, USA) to assess the impact of peptides on cell proliferation.

### 2.8. Antimicrobial Assays of Peptides

The minimum inhibitory concentration (MIC) and minimum bactericidal concentration (MBC) of peptides against *Staphylococcus aureus* NCTC 10788, *Escherichia coli* ATCC 8739, *Candida albicans* NCTC 10231, and MRSA NCTC 12493 were detected to evaluate the antimicrobial ability of peptides. The approach was consistent with previous studies [18]. First, the microorganisms were cultured in Mueller Hinton Broth (MHB) for 18 h. Different concentrations of peptides were mixed with microbial solutions in the logarithmic growth phase. After overnight incubation, the lowest concentration that inhibited bacterial growth was MIC and the lowest peptide concentration corresponding to no colony growth in MHA-containing dishes was MBC.

### 2.9. Smooth Muscle Pharmacological Activity Assay of Peptides

As in the previous smooth muscle tone assay [11], male Wistar rats were euthanized by carbon dioxide asphyxiation, according to the ethical permission of animal experiments. Smooth muscles of the bladder, ileum, artery, and uterus were removed from the rat, dissected into strips, and stretched in the 2 mL organ bath containing Krebs solution under 95% O_2_, and 5% CO_2_ perfusion. The basal muscle tension was set to 0.5 g. The peptide was dissolved in Krebs solution to form the peptide solution of 10^−5^ to 10^−9^ M. Between different concentrations, the muscles were washed for 5 min and equilibrated for 5 min. The peptide solution was then added to the organ bath every 10 min to observe the peptide change in smooth muscle tone. Changes in muscle tone were recorded by pressure sensors of the Power Lab system (AD Instruments Pty Ltd., Oxford, UK).

To study the ion pathways of peptides that affect smooth muscle tone, 20 µL of potassium ion channel blocker aminopyridine (10^−3^ M) was added 10 min before the peptide was injected into the organ bath. The subsequent steps were the same as the smooth muscle tone assay.

To examine the effect of Senegalin-2BK on bradykinin, 20 μL 10^−7^ M Senegalin-2BK was added 10 min before administering bradykinin into the bladder organ bath. The following steps were consistent with the smooth muscle tone assay. The dose–response curves were plotted from the muscle tone data, and the mean and standard errors of the data were analyzed by the Student’s *t*-test.

### 2.10. Molecular Simulation of Peptide and Its Molecular Docking

The binding mode of the bradykinin (BK) antagonist, Senegalin-2BK, with the BK receptor (B2R), was explored through peptide structure simulation and receptor docking, hypothesizing its antagonistic effect via interaction with B2R.

The peptide structure was constructed using ChemBioDraw Ultra 14.0 and subsequently subjected to energy minimization in ChemBio3D Ultra 14.0, with the minimum RMS gradient set at 0.001. The optimized peptide structure was then saved in ProteinData Bank (PDB) format. The structure of the B2R (PDB ID: 7F20) was downloaded from the PDB database. Prior to docking, crystallographic waters and original ligands were removed from the protein structure using Pymol 2.3.0. Both Senegalin-2BK and B2R structures were uploaded to HPEPDOCK 2.0 for molecular docking, and the interaction mode was visualized by Pymol 2.3.0.

## 3. Results

### 3.1. “Shotgun” Cloning of Senegalin-2 Precursor-Encoding cDNAs from the Kassina senegalensis cDNA Library

**Figure 1:**
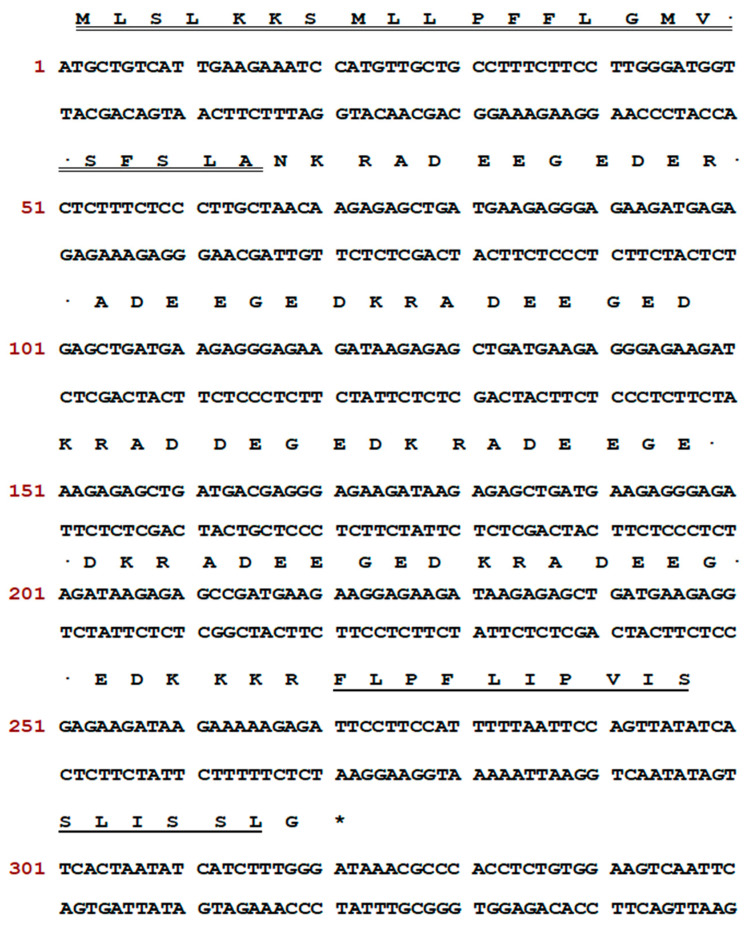
The nucleotide sequence and translated amino acid sequence of Senegalin-2. Signal peptide, mature peptide, and terminal codon are marked by double underline, single underline, and asterisk, respectively.

The precursor nucleotide sequence and the corresponding translated amino acid sequence of the encoded peptide Senegalin-2 from the skin secretion-derived cDNA library are presented in Figure 1. The open reading frame first presented a highly conserved signal peptide with an acidic amino acid-rich spacer region in the middle, ending with the mature peptide sequence consisting of 16 amino acids. The N-terminus of Senegalin-2 was the cleavage-specific protein convertase processing site “-lysine-arginine-” (-K-R-), and its C-terminus was amidated with glycine as the donor.

### 3.2. Sequence Characterization and Comparison of Senegalin-2

**Figure 2:**
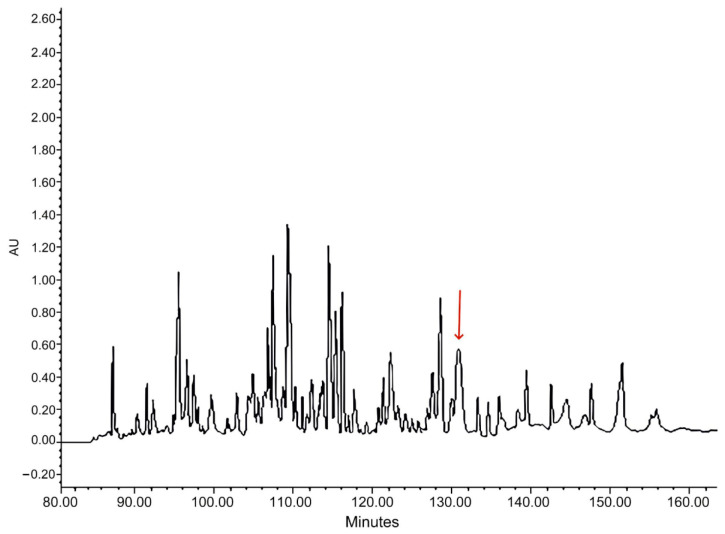
The RP-HPLC chromatogram of crude extracts from *Kassina senegalensis* frog skin secretion detected at 214 nm. The peak containing Senegalin-2 is marked with the red arrow, and its retention time is 131 min.

**Figure 3:**
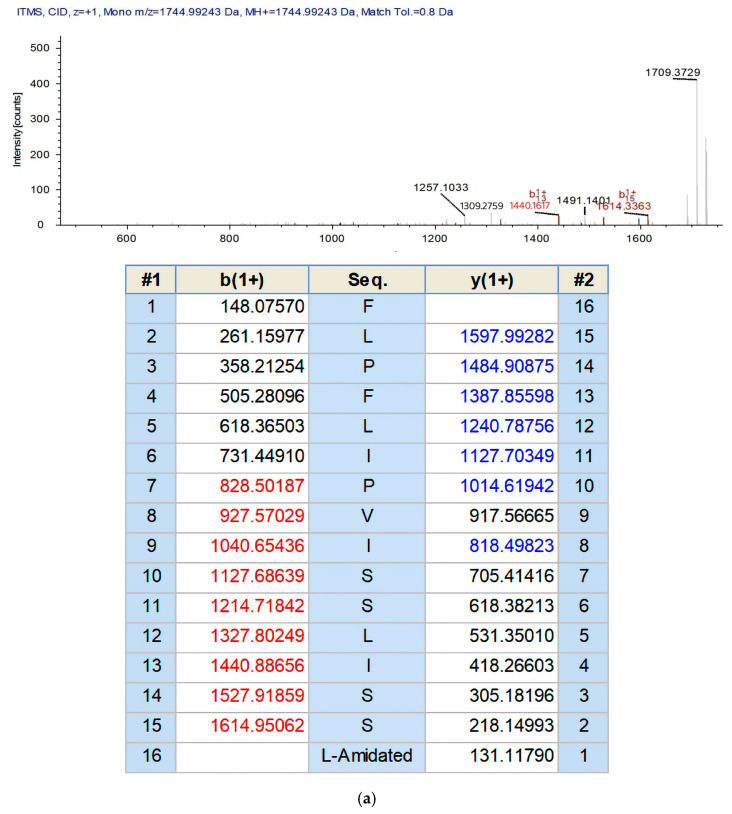

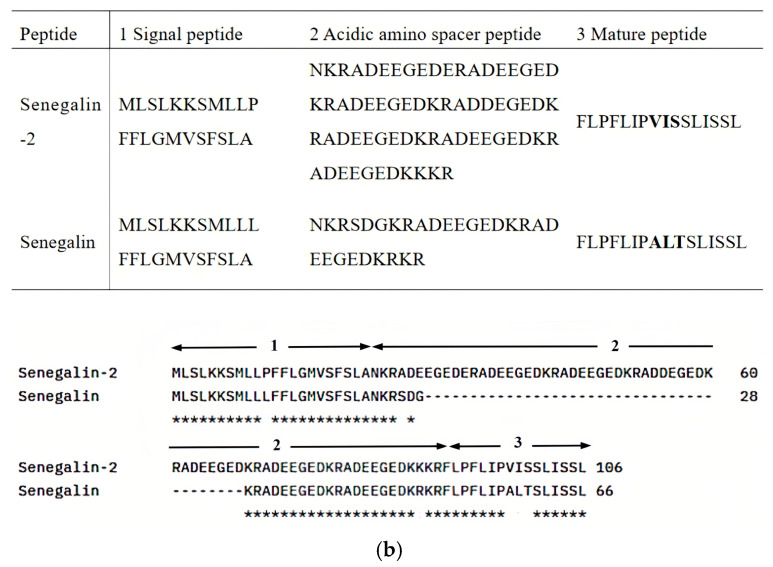
(**a**) The b and y ions of the amino acids contained in Senegalin-2 were detected in the mass spectrometry, and are marked in red and blue, respectively. Seq represents the sequence of Senegalin-2. (**b**) Sequence comparison of Senegalin-2 with Senegalin. The signal peptide region is labeled 1, the acidic spacer region is 2, and the mature peptide region is 3. Asterisks indicate common amino acid residues.

The *Kassina senegalensis* skin secretions were separated by RP-HPLC (Figure 2). The sequence of Senegalin-2, Phe-Leu-Pro-Phe-Leu-Ile-Pro-Val-Ile-Ser-Ser-Leu-Ile-Ser-Ser-Leu-amide (FLPFLIPVISSLISSL-NH_2_), was determined by tandem mass spectrometry (MS/MS) (Figure 3a).

From the sequence alignment results, Senegalin-2 showed high similarity to the reported peptide Senegalin from the same species (Figure 3b). The result suggest that these peptides are homologous and can be classified into the same peptide family.

### 3.3. Fusion Peptide Design

In previous studies, certain amphibian myotropic peptides belonging to the bradykinin-related peptides (BRPs) were found to antagonize bradykinin (BK), with their peptide chains containing the BK sequence. Based on this, we adopted a mimicry strategy to fuse bioactive peptides with BK, aiming to design BK antagonists with multiple pharmacological activities.

When selecting peptides to conjugate with BK for antagonist construction, we focused on both myotropic effects and antimicrobial activity. Firstly, peptides exhibiting opposite myotropic effects to BK were screened to ensure the antagonistic efficacy of the fusion peptide. Senegalin-2 was chosen due to its relaxing effect on rat bladder smooth muscle, while peptides such as Senegalin, Kassorin, and WL-8 were excluded because they shared similar myotropic effects with BK (bladder contraction and artery relaxation). Secondly, considering the therapeutic challenges posed by concomitant infections, we prioritized peptides with antimicrobial properties. Senegalin-2 demonstrated significant antibacterial activity against Gram-positive bacteria (MIC 2 μM), making it more suitable than Senegalin (MIC 50 μM) with weaker activity and Kassorin M with no antibacterial capability. Therefore, Senegalin-2 emerged as an ideal candidate peptide for constructing the BK antagonist.

After identifying Senegalin-2 as the candidate peptide, we synthesized the fusion peptide Senegalin-2BK by linking it to the BK sequence via a triglycine (GGG) linker (Table 1). This design was intended to leverage the flexibility and stability of the GGG linker to enhance the antagonist’s efficacy. Additionally, we anticipate that Senegalin-2BK will retain the antimicrobial activity of Senegalin-2, thus possessing the potential to counteract BK while also defending against microbial invasion.

The purification liquid chromatograms and mass spectra of Senegalin-2 and Senegalin-2BK are in Figures S1 and S2, respectively.

### 3.4. Physicochemical Properties and Structure Prediction of Senegalin-2 and Senegalin-2BK

**Figure 4:**
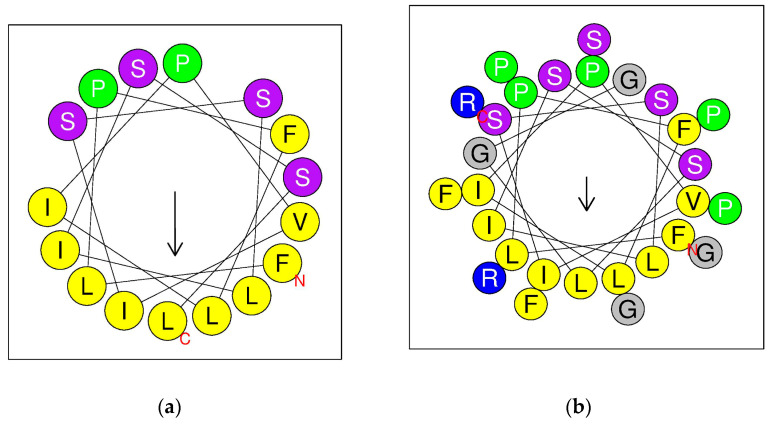
Helical wheel diagrams of Senegalin-2 (**a**) and Senegalin-2BK (**b**) and color-coding amino acid residues based on their properties: positively charged (blue), hydrophobic (yellow), uncharged polar (purple), aromatic (green), and other non-polar/glycine (grey).The red C and N represent the C-terminus and N-terminus of the peptide chain, respectively. The arrows indicate the direction of the hydrophobic moment.

**Figure 5:**
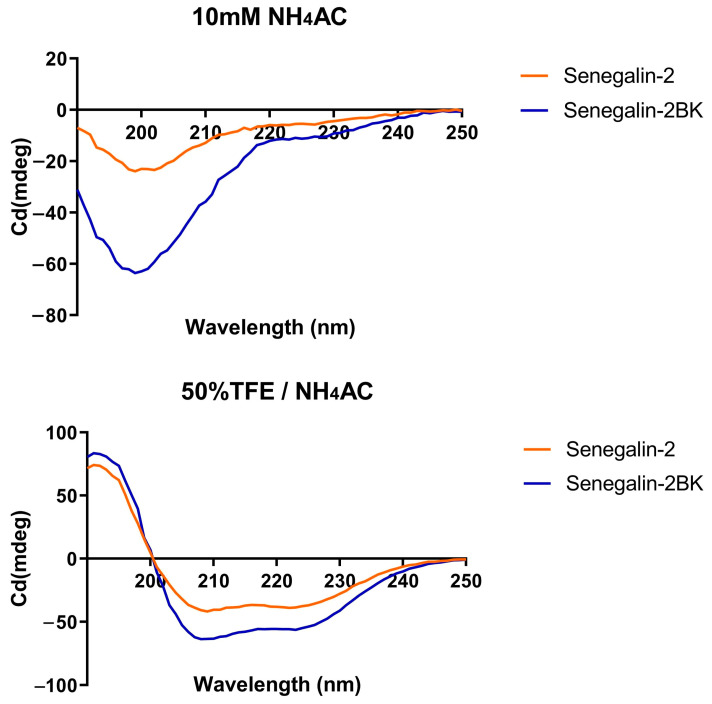
The secondary structure determination of Senegalin-2 and Senegalin-2BK. The peptide solution was dissolved in 10 mM NH_4_Ac solution (pH 7.4) and 50% TFE/10 mM NH_4_Ac solution (pH 7.4) in 50 µM for CD.

The amino acid composition and hydrophobic moment of Senegalin-2 and Senegalin-2BK are visualized in Figure 4, highlighting differences in their physicochemical properties (Table 2). Compared to Senegalin-2, Senegalin-2BK exhibited reduced hydrophobicity, increased positive charge, an additional hydrophobic surface (VPFGLLGLIF), and decreased helix ratio. The CD spectroscopy revealed that both peptides adopted α-helix conformations in simulated microbial membranes, indicated by peaks at 192, 208, and 222 nm, whereas they appeared disordered in a water-mimicking environment (peak at 207 nm) (Figure 5).

### 3.5. Hemolytic Activity and Cytotoxicity of Senegalin-2 and Senegalin-2BK

**Figure 6:**
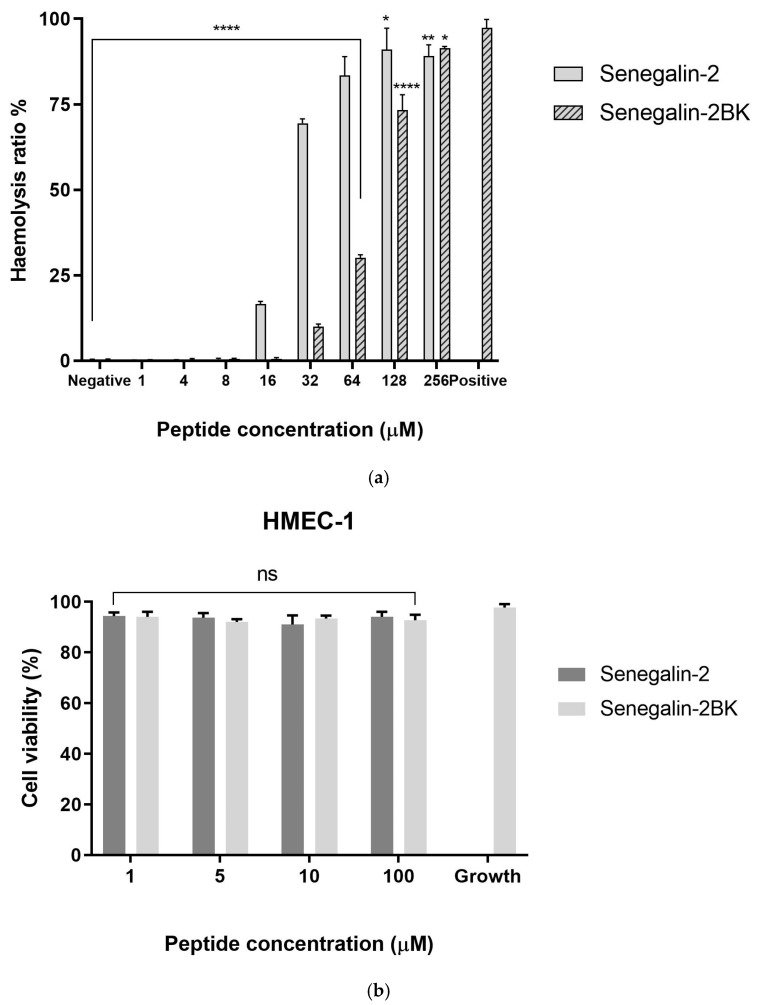
(**a**) The hemolytic assay of Senegalin-2 and Senegalin-2BK on horse erythrocytes. Two-way ANOVA compared the results of the peptide group and the positive control group. The HC_50_ values of peptides on erythrocytes are in Table 3. (**b**) The effect of the peptides on the proliferation of normal cell lines HMEC-1. The cell viability (%) is applied to reflect peptide inhibition of cell lines. Two-way ANOVA compared the results of the peptide group and the growth control group. The error bars indicate the mean ± SEM of three replicates, ns = no significant difference, * = *p* < 0.05, ** = *p* < 0.01, and **** = *p* <0.0001.

The hemolytic activities and cytotoxicity of the peptides are shown in Figure 6a and 6b, respectively. For Senegalin-2, a marked elevation in the hemolysis ratio was observed at 32 μM, exceeding 50%. Senegalin-2BK induced over 25% hemolysis at 64 μM. Table 3 summarizes the half-maximal hemolytic concentrations (HC_50_) for the peptides, indicating that Senegalin-2BK (HC_50_ = 85.09 μM) is less prone to inducing erythrocyte lysis compared to Senegalin-2 (HC_50_ = 23.19 μM). In the MTT assay, both peptides exhibited no cytotoxicity towards the HMEC-1 cell line.

### 3.6. Antimicrobial Activity of Senegalin-2 and Senegalin-2BK

As demonstrated in Table 4, both Senegalin-2 and Senegalin-2BK exhibited strong bactericidal activity against *S. aureus* and MRSA, with a MIC of 2 μM and achieving lethality at concentrations not exceeding 4 μM. A notable distinction is that Senegalin-2 is ineffective against *E. coli*, whereas Senegalin-2BK demonstrated bactericidal action against *E. coli* at 128 μM. The selectivity index (SI) values of Senegalin-2 and Senegalin-2BK against various microorganisms are in Table 5. The SI values of Senegalin-2 against *S. aureus* and MRSA were determined to be 11.6, whereas the SI of Senegalin-2BK increased to 42.55 for these two Gram-positive bacteria and was measured at 21.27 for *E. coli*.

### 3.7. Pharmacological Effects of Senegalin-2 and Senegalin-2BK on Rat Smooth Muscle Preparations

**Figure 7:**
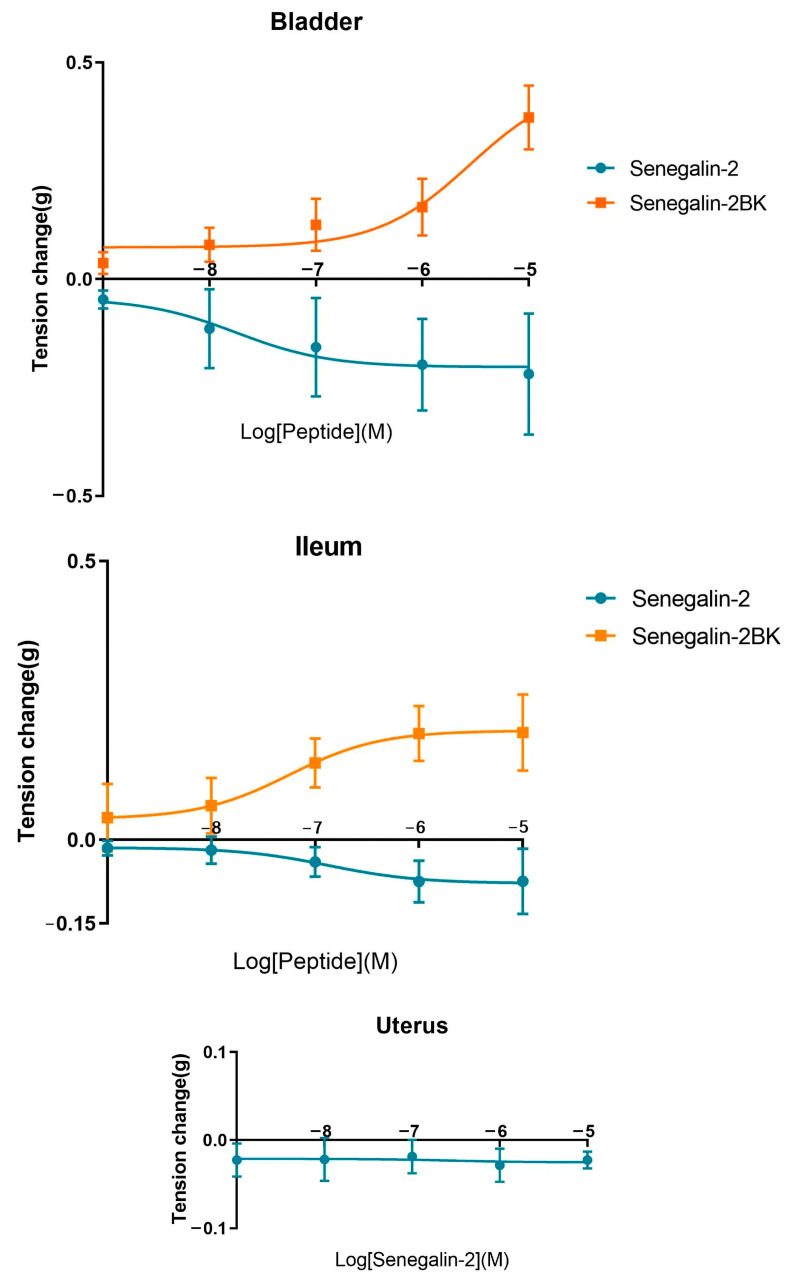

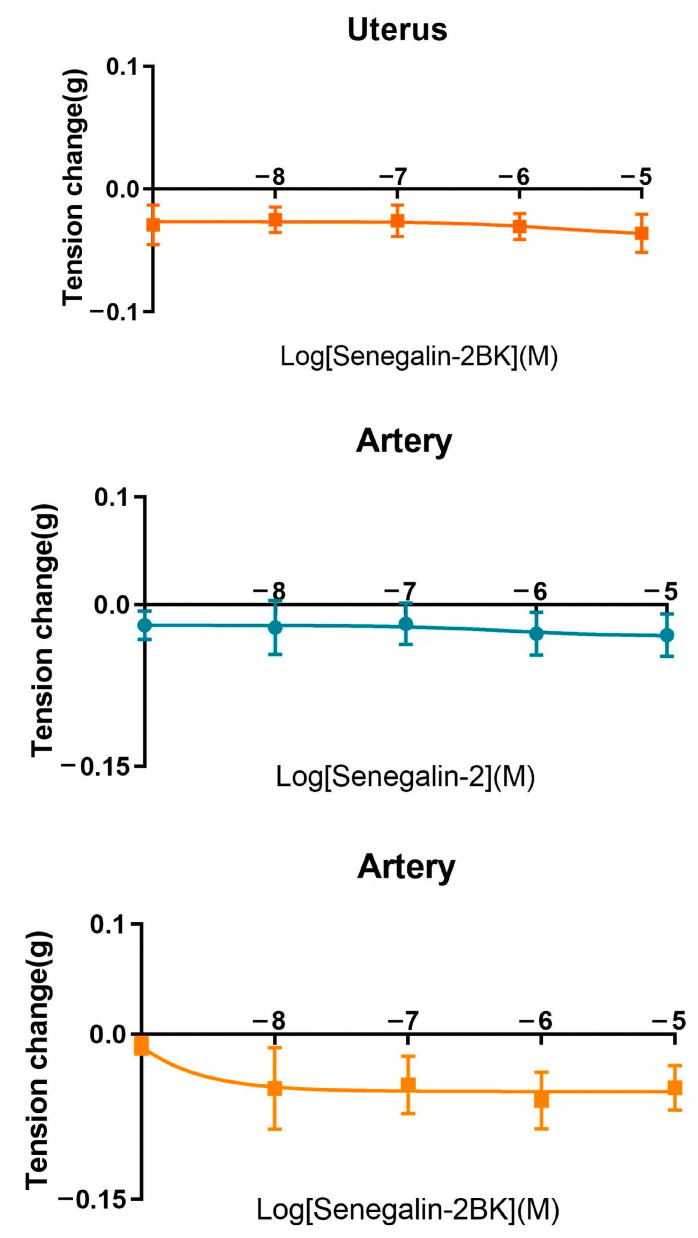
The dose–response curves of Senegalin-2 and Senegalin-2BK to smooth muscle tension changes by applying isolated smooth muscle preparations of rat bladder, uterus, artery, and ileum. Error bars indicate the SEM of the mean of the data obtained from three independent experiments.

The pharmacological activities of Senegalin-2BK on the smooth muscle of the rat bladder, uterus, artery, and ileum, and its comparison with Senegalin-2, are show in Figure 7. Senegalin-2 effectively relaxed the bladder smooth muscle, exhibiting an EC_50_ of 17.94 nM, and also induced relaxation in the ileum with an EC_50_ of 135 nM, while demonstrating minimal effects on both arteries and the uterus. Unlike Senegalin-2, Senegalin-2BK induces contraction in the rat’s bladder and ileum, with EC_50_ values of 2.84 μM and 56.64 nM, respectively. Both peptides have negligible effects on artery and uterus smooth muscles.

**Figure 8:**
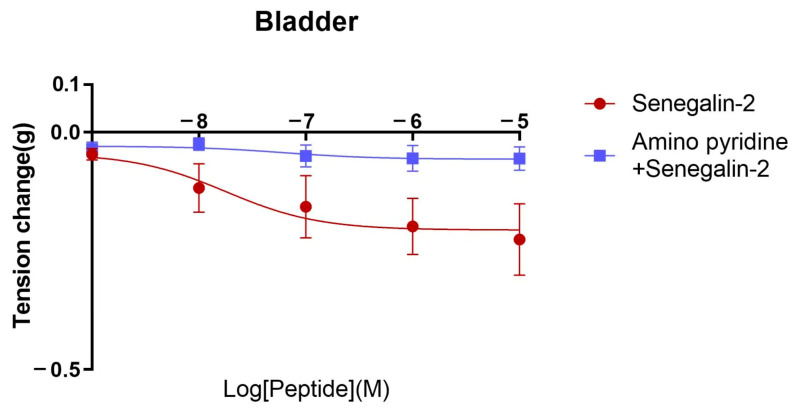

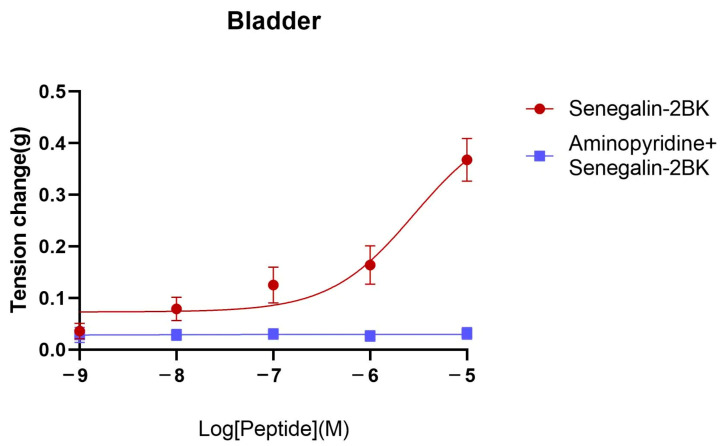
The relaxation effect of Senegalin-2 and Senegalin-2BK on rat bladder smooth muscle after pre-adding potassium channel blocker-amino pyridine. Amino pyridine (10^−3^ M) was selected to pre-impact the bladder smooth muscle. Error bars indicate the SEM of the mean of the data obtained from three independent experiments.

The effect of the two peptides on bladder smooth muscle and the alteration of their effects after the pre-addition of potassium channel blocker-amino pyridine are shown in Figure 8. As illustrated in the graphs, amino pyridine inhibited the relaxation of the bladder smooth muscle activity of both two peptides. The effect of Senegalin-2 and Senegalin-2BK was attenuated more significantly by amino pyridine at 10^−5^ M. These data suggest that Senegalin-2 and Senegalin-2BK may regulate the tension of bladder smooth muscle through potassium channels.

**Figure 9:**
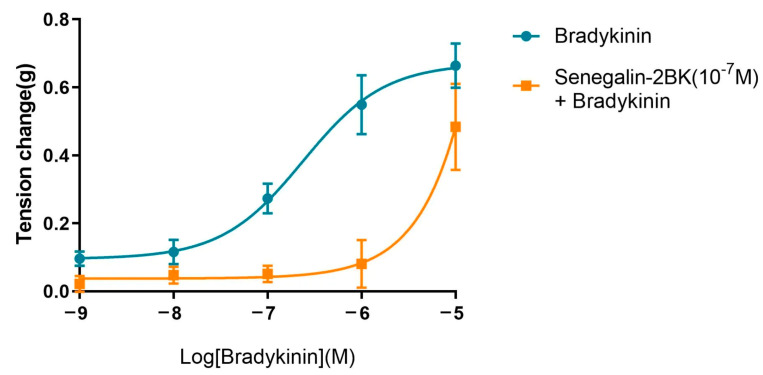
The dose–response curves of bradykinin-induced bladder smooth muscle contraction with or without the pre-addition of Senegalin-2BK. The dose–response curve of bradykinin (sequence: RPPGFSPFR) on rat bladder smooth muscle, marked in blue, exhibited an EC_50_ of 2.38 × 10^−7^ M. After pre-incubation with 10^−7^ M Senegalin-2BK, the dose–response curve of bradykinin on rat bladder smooth muscle is shown in orange. Error bars represent the standard error of the mean (SEM) data obtained from three independent experiments.

Moreover, the effects of Senegalin-2BK on the activities of Bradykinin (sequence: RPPGFSPFR) on rat’s bladder smooth muscle were tested. Bradykinin induced contraction of rat bladder smooth muscle with an EC_50_ of 2.38 × 10^−7^ M. After pre-incubation with 10^−7^ M Senegalin-2BK, the bladder smooth muscle contraction induced by bradykinin was inhibited (Figure 9). The suppressing effect of Senegalin-2BK increased with bradykinin concentration from 10^−6^ M to 10^−9^ M, but significantly weakened when bradykinin exceeded 10^−6^ M.

### 3.8. Molecular Simulation of Senegalin-2BK and Its Docking with Bradykinin B2 Receptor (B2R)

**Figure 10:**
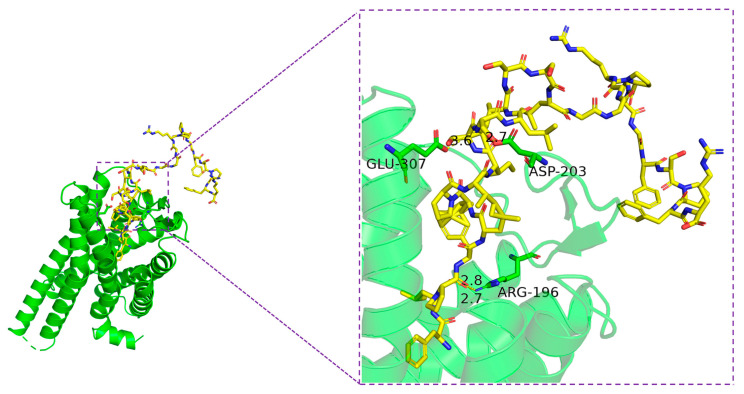
Intermolecular interactions between Senegalin-2BK and the bradykinin B2 receptor (B2R). The backbone of Senegalin-2BK is in yellow, with oxygen atoms in red and nitrogen atoms in blue. The green helical structure is the main body of B2R. The residues of B2R involved in binding are shown as sticks.

As presented in Figure 10, Senegalin-2BK reflected favorable binding affinity to B2R with a binding energy of −273.333 kcal/mol. It formed hydrogen bonds with Arginine (Arg)-196, Aspartic Acid (Asp)-203, and Glutamic acid (Glu)-307 of B2R, with bond lengths of 2.8 Å, 2.7 Å, 2.7 Å, and 3.6 Å, respectively. These residues played important roles in the interaction between Senegalin-2BK and B2R.

## 4. Discussion

Amphibian defensive peptides are natural compounds that play a crucial role in the physiological activities related to host immunity [20]. As a type of defensive peptide, amphibian myotropic peptides play a crucial role in host neuromodulation and predator avoidance [21,22]. Meanwhile, some amphibian myotropic peptides inhibited the pharmacological activity of bradykinin (BK), making them lead compounds for treating diseases associated with BK overexpression [10,11,23]. This offered hope for alleviating the shortage of clinical BK inhibitors. However, their efficacy was relatively low, and their safety as drugs remained unclear.

The *Kassina* frog is a source of myotropic peptides with special structures and abundant activities. Here, Senegalin-2, a peptide discovered in the *Kassina senegalensis* frog, demonstrated potent antibacterial activity against Gram-positive bacteria and induced relaxation of both bladder and ileum by modulating potassium channels. According to the structural characteristics of BK antagonists, the BK inhibitor Senegalin-2BK was artificially designed based on Senegalin-2. Senegalin-2BK exhibited a higher selectivity index for antimicrobial activity and reduced susceptibility to causing hemolysis. Muscle mechanics assays proved that 10^−7^ M Senegalin-2BK has no toxicity and effectively inhibited BK-induced contraction of rat bladder smooth muscle. Furthermore, Senegalin-2BK exerted contractile effects on the rat bladder and ileum smooth muscles.

The natural peptide Senegalin-2 induced a relatively higher proportion of hemolysis. However, in the smooth muscle tension experiments, it did not induce erythrocyte rupture at therapeutic concentrations. Moreover, it exhibited no cytotoxicity towards normal vascular endothelial cells. Compared to Senegalin-2, the HC_50_ value of Senegalin-2BK was significantly reduced due to its lower hydrophobicity and the appropriate introduction of cationic charges. The alteration of physicochemical properties is a crucial determinant of which peptides lyse red blood cells [24,25]. If Senegalin-2BK progresses to the clinical drug development stage in the future, systemic toxicity assessments will be required. Combining the MIC from antibacterial assays with the HC_50_ from hemolysis assays, we calculated the SI values. A high SI indicates favorable safety and efficacy profiles for the compounds. Compounds with an SI ≥ 10 are considered to have the potential for further development [19,26]. The SI of Senegalin-2 against Gram-positive bacteria was greater than 10, qualifying it as a lead compound with good selectivity. Senegalin-2BK exhibited an SI exceeding 40 against S. aureus and MRSA and an SI greater than 20 against *E. coli*, classifying it as a highly efficient and low-toxicity potential bioactive peptide.

In pharmacological activity assays, compared to the reported peptide Senegalin (*S. aureus* MIC 50 μM), Senegalin-2 demonstrated stronger antibacterial effects (*S. aureus* MIC 2 μM) and its myotropic effect was opposite to that of Senegalin, causing relaxation of rat bladder and ileum smooth muscles with the EC_50_ of 17.94 nM and 135 nM, respectively. Generally, the effectiveness of antibacterial peptides is closely related to parameters such as positive charge, hydrophobicity, and helical structure [27]. Both Senegalin-2 and Senegalin are uncharged, yet they exhibit inhibition against Gram-positive bacteria. This may be attributed to the hydrophobicity and α-helical structure of these peptides, which facilitate the binding of the peptide chains to microbial phospholipid layers. However, their high hydrophobicity may also hinder their ability to penetrate the outer membrane of Gram-negative bacteria, explaining their ineffectiveness against *E. coli* [28,29]. Senegalin-2 and Senegalin share a high sequence similarity, differing only in the amino acids at positions 8 to 10. However, Senegalin-2, containing Val8, Ile9, and Ser10, demonstrates amplified antibacterial effects and an alteration of smooth muscle tone activity. This demonstrates that amino acid substitutions can ultimately modulate peptides’ efficacy [7,30,31]. Thus, an active motif, “valine-isoleucine-serine”, can be proposed for subsequent modification studies of bioactive peptides.

The hydrophobicity of the modified peptide Senegalin-2BK is lower than that of Senegalin-2, but it possesses a complete hydrophobic surface and two additional positive charges compared to the natural peptide Senegalin-2. On the one hand, the hydrophobic surface of Senegalin-2BK will bind to the hydrophobic lipid core, inducing the insertion of the peptide into the microbial cell membrane lipid bilayer, causing pore formation and ultimately killing the microorganism [32]. On the other hand, the guanidine groups carried by the arginines in Senegalin-2BK confer cationic properties to the peptide, facilitating interaction with the anion-rich bacterial membrane [33]. Therefore, Senegalin-2BK achieves a better balance of physicochemical properties, maintaining efficacy against Gram-positive bacteria while inhibiting Gram-negative bacteria, broadening its antibacterial spectrum.

In the screening process for myotropic peptide activity, the novel natural peptide Senegalin-2 had relaxant effects on rat bladder smooth muscle, highlighting its potential as a therapeutic agent for the treatment of overactive bladder. Overactive bladder (OAB) is a condition typified by urgency, frequency, and involuntary urine leakage, often ascribed to heightened detrusor activity during bladder filling [34]. Traditional treatments for OAB primarily rely on anticholinergic drugs, but unfortunately, these medications can lead to side effects such as dry mouth and palpitations [35]. Additionally, pathogenic bacteria are commonly found in the urine of OAB patients [36]. Senegalin-2 offers a dual advantage: it addresses urinary tract infections (UTI) and circumvents the aforementioned adverse reactions, alleviating bladder muscle tension, thereby being considered a promising natural treatment candidate for OAB. The fusion peptide Senegalin-2BK exhibits contractile effects on rat bladder and ileum smooth muscle. This suggests its potential as a lead compound for treating urinary retention and constipation [37,38]. These conditions typically arise from bladder contraction disorders and reduced intestinal motility. Specifically, the typical treatment for urinary retention involves indwelling catheters. However, a frequent side effect of this relief method is the development of UTI, leading to severe complications [39]. Senegalin-2BK holds promise in restoring normal organ contractile function while inhibiting microbial proliferation within catheters, either through coating modifications or direct topical application, providing potential therapeutic options for these common ailments.

Our studies indicate that the two peptides’ effects on bladder relaxation are inhibited by aminopyridine, an effective inhibitor of voltage-gated potassium channels (Kv) [40,41]. This suggests that Kv channels play a crucial role in mediating peptides’ action on the bladder. These findings provide valuable insights into the mechanism behind peptides’ therapeutic effects and pave the way for further clinical investigations.

In the smooth muscle assay, low concentrations of Senegalin-2BK significantly inhibited the effects of BK, primarily attributed to its design. One notable feature of this design is the synthesis of Senegalin-2BK through the linkage of two peptides via a glycine-rich flexible linker (GGG). This GGG linker enables each domain to maintain its distinct functionality while imparting additional derivative activity. Consequently, Senegalin-2BK combined both peptides’ advantages and exhibited antagonistic effects against BK [42,43,44]. Another key design aspect is the artificial synthesis of Senegalin-2BK based on the sequence characteristics of bradykinin-related peptides (BRP). The experimental results validate our concept of designing BRP to synthesize BK antagonist peptides.

Bradykinin is known to activate nociceptors under pathological conditions, leading to symptoms such as pain and hypersensitivity [45,46]. The bradykinin B2 receptor (B2R) is closely associated with these symptoms. In recent years, B2R emerged as an ideal target for developing BK antagonist drugs [47]. According to molecular docking results, Senegalin-2BK showed high binding specificity and affinity towards B2R (Figure 10). This specificity facilitates the selective targeting of B2R by Senegalin-2BK in biological systems, highlighting its potential as a B2R antagonist. Additionally, Senegalin-2BK formed hydrogen bonds with specific residues of B2R, which contribute to stabilizing the peptide–receptor complex and participating in the interaction. These findings provide preliminary insights into the molecular mechanism underlying the antagonistic effects of Senegalin-2BK against BK.

As a BK antagonist with antibacterial properties, Senegalin-2BK holds promise for exhibiting therapeutic potential in maintaining blood pressure stability and reducing inflammation. This fusion peptide may serve as an inspiration for the structure of new BK antagonist drugs [48].

## 5. Conclusions

This study successfully isolated a bioactive peptide, Senegalin-2, from the *Kassina* frog. This peptide demonstrates notable antibacterial properties and a relaxing effect on bladder smooth muscle. Moreover, we developed Senegalin-2BK, a fusion peptide with enhanced antibacterial activity, myotropic effects, and BK antagonistic capabilities. This achievement marks a significant advancement in exploring peptide resources derived from amphibians and provides a promising foundation for developing peptide-based bradykinin antagonists. Several critical steps must be undertaken to facilitate the clinical translation of these promising peptides. It is essential to assess their pharmacokinetic profiles, which includes evaluating absorption, distribution, and metabolism. This information will be instrumental in optimizing dosage and determining appropriate administration routes. We also need to evaluate the sensitivity of these peptides to proteolytic enzymes to identify potential modifications that could enhance their stability and efficacy in vivo. Additionally, a comprehensive investigation of the receptor–ligand mechanisms involving Senegalin-2B as a BK antagonist is crucial for understanding the interactions between Senegalin-2BK and its target receptor. Furthermore, we should explore the therapeutic potential of Senegalin-2 and Senegalin-2BK for overactive bladder and urinary tract infections and their roles in combination therapies.

Overall, the introduction of these peptides into clinical practice necessitates preclinical evaluation, safety assessments, and clinical trials. Our ultimate objective is to develop a series of safe and effective therapeutic agents based on Senegalin-2 and Senegalin-2BK to address muscle-related clinical conditions.

## Figures and Tables

**Table 1:** Amino acid sequence of Bradykinin, Senegalin-2, and the fusion peptide Senegalin-2BK.

Peptide	Mature Peptide Sequence
Bradykinin	RPPGFSPFR
Senegalin-2	FLPFLIPVISSLISSL-NH_2_
Senegalin-2BK	FLPFLIPVISSLISSLGGGRPPGFSPFR-NH_2_

**Table 2:** Physicochemical properties of peptides. The α-helix (%) of the peptides was calculated using the online software Bestsel (v1.3.230210) (https://bestsel.elte.hu/index.php, accessed on 25 May 2024) based on their CD spectra.

Peptide (Sequence)	Hydrophobicity <H>	Hydrophobic Moment <µH>	Net Charge	α-Helix (%)
Senegalin-2 (FLPFLIPVISSLISSL-NH_2_)	1.143	0.491	0	67.2
Senegalin-2BK (FLPFLIPVISSLISSLGGGRPPGFSPFR-NH_2_)	0.784	0.315	+2	55.7

**Table 3:** The HC_50_ value of peptides on horse erythrocytes.

Peptide	HC_50_ (μM)
Senegalin-2	23.19
Senegalin-2BK	85.09

**Table 4:** The MIC and MBC of Senegalin-2 and Senegalin-2BK against microorganisms.

MIC/MBC (µM)				
Peptide	*S. aureus* NCTC 10788	*E. coli* ATCC 8739	*C. albicans* NCTC 10231	MRSA NCTC 12493
Senegalin-2	2/2	>128/>128	>128/>128	2/4
Senegalin-2 BK	2/2	64/128	>128/>128	2/4

**Table 5:** The SI values of Senegalin-2 and Senegalin-2BK on microorganisms.

SI ^1^				
Peptide	*S. aureus* NCTC 10788	*E. coli* ATCC 8739	*C. albicans* NCTC 10231	MRSA NCTC 12493
Senegalin-2	11.6	- ^2^	-	11.6
Senegalin-2 BK	42.55	21.27	-	42.55

^1^ The selective index (SI) is determined as the ratio of of HC50 (µM) to the MIC (µM) [19]. ^2^ When the MIC exceeded 128 µM, the SI value was not included in the calculation and is denoted as “-”.

## Data Availability

The cDNA encoding the biosynthetic precursor of Senegalin-2 has been deposited in the NCBI Database with the accession number PQ790043.

## Supplementary Materials

The following supporting information can be downloaded at: https://www.mdpi.com/article/10.3390/biom15010030/s1, Figure S1: The RP-HPLC chromatograms of Senegalin-2 (a) and Senegalin-2BK (b); Figure S2: The mass spectrum of the peptides (Senegalin-2 (a), Senegalin-2BK (b)).
