# Supplementary material for: Senegalin-2: A Novel Hexadecapeptide from Kassina senegalensis with Antibacterial and Muscle Relaxant Activities, and Its Derivative Senegalin-2BK as a Bradykinin Antagonist
Source: Biomolecules. 2024 Dec 30;15(1):30. doi: 10.3390/biom15010030 (PMC11764382; doi:10.3390/biom15010030)
Supplement: Supplementary file 1 [file biomolecules-15-00030-s001.zip › Supplementary Materials.pdf]

Supplementary Materials

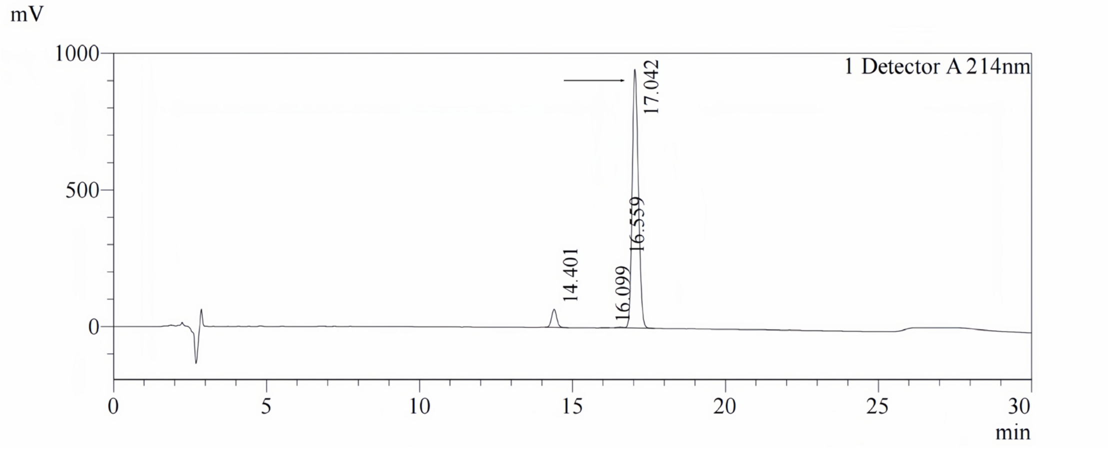

(a)

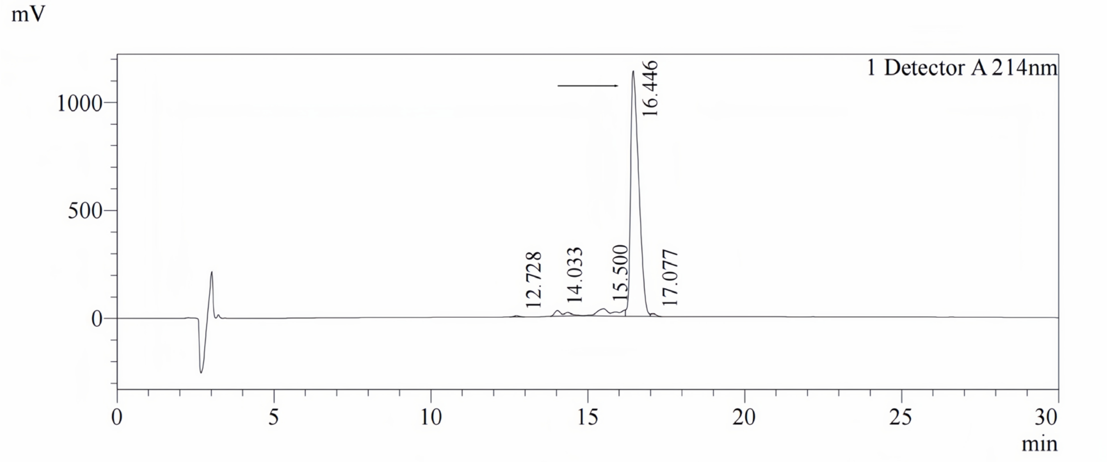

(b)

Figure S1 The RP-HPLC chromatograms of Senegalin-2 (a) and Senegalin-2BK (b). The peaks marked by the arrow were the peptides that exhibited more th an 95% purity, and the numbers in the graph were the retention times of the component.

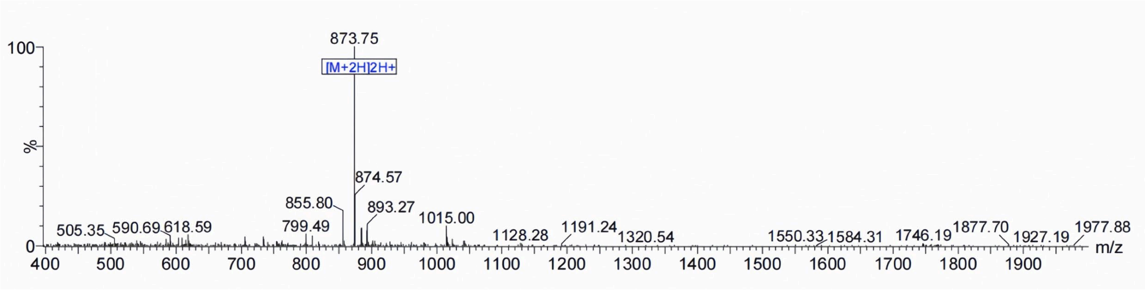

(a)

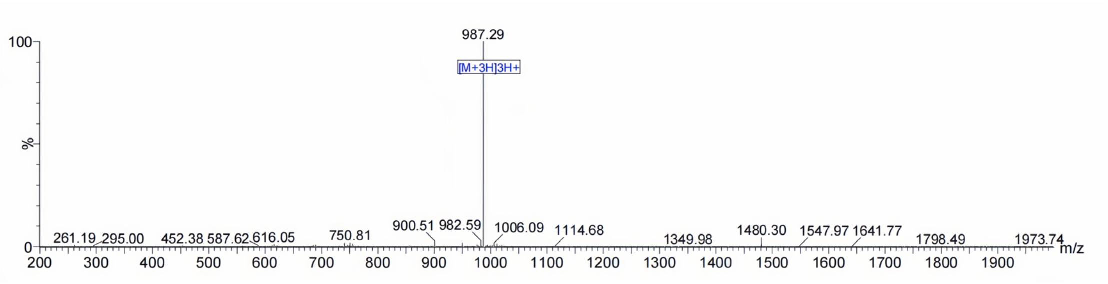

(b)

Figure S2 The mass spectrum of the peptides (Senegalin-2(a), Senegalin-2BK (b)). The [M+2H]2H+ mass-to-charge ratio (m/z) ion peak in Figure S2 (a) wa s +2 charged Senegalin-2 carrying 2 hydrogen ions. The [M+3H]3H+ m/z ion peak was +3 charged Senegalin-2BK with 3 hydrogen ions. The measured mol ecular mass of Senegalin-2 (a), Senegalin-2BK (b) was 1745 and 2958, which was consistent with the sequence calculations.
