# Supplementary figures and images for: Senegalin-2: A Novel Hexadecapeptide from Kassina senegalensis with Antibacterial and Muscle Relaxant Activities, and Its Derivative Senegalin-2BK as a Bradykinin Antagonist
Source: Biomolecules. 2024 Dec 30;15(1):30. doi: 10.3390/biom15010030 (PMC11764382; doi:10.3390/biom15010030)

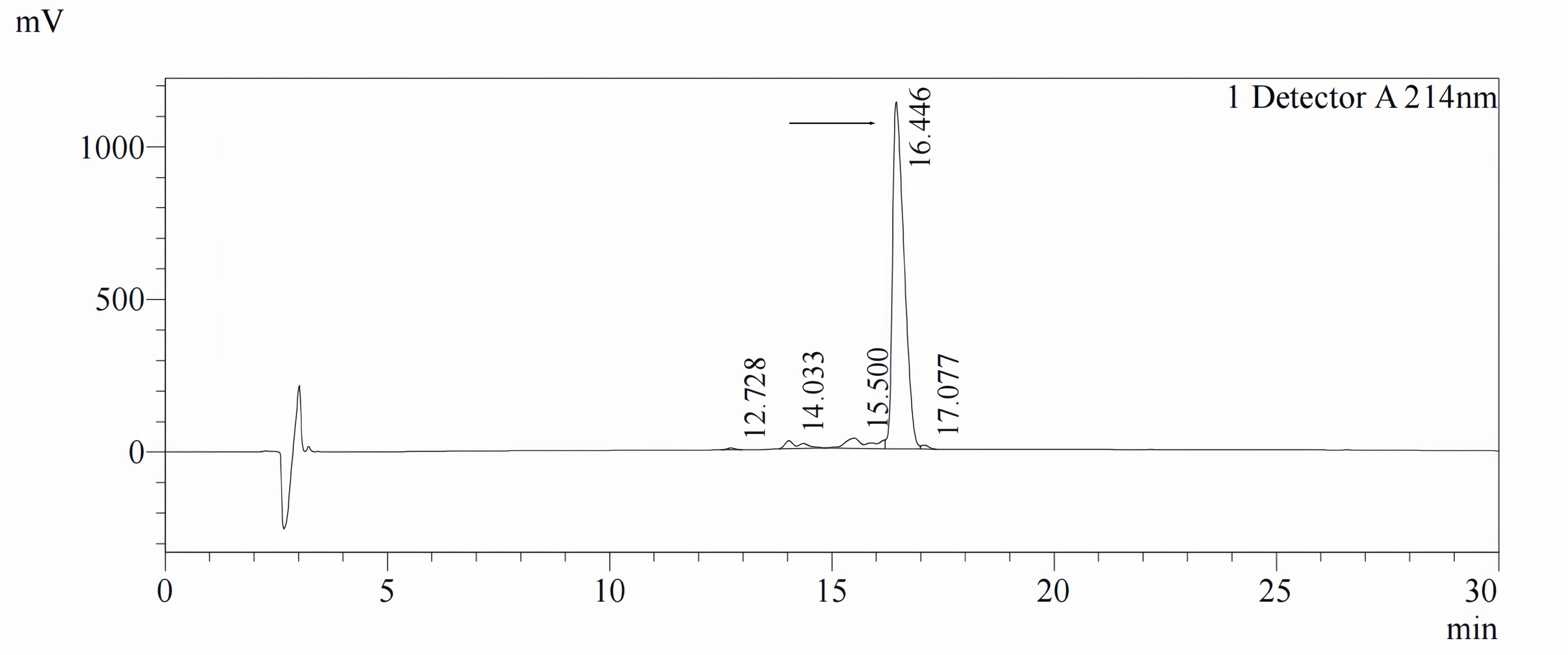

Supplement: Supplementary file 1 [file biomolecules-15-00030-s001.zip › Segalin-2BKhplc.jpg]

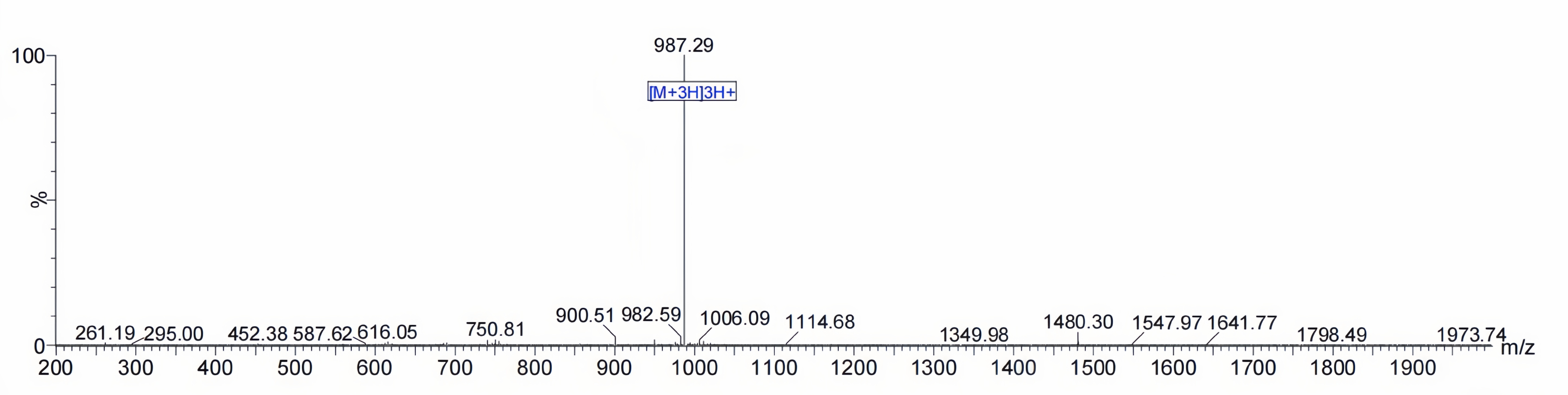

Supplement: Supplementary file 1 [file biomolecules-15-00030-s001.zip › Senegalin-2BKms.jpg]

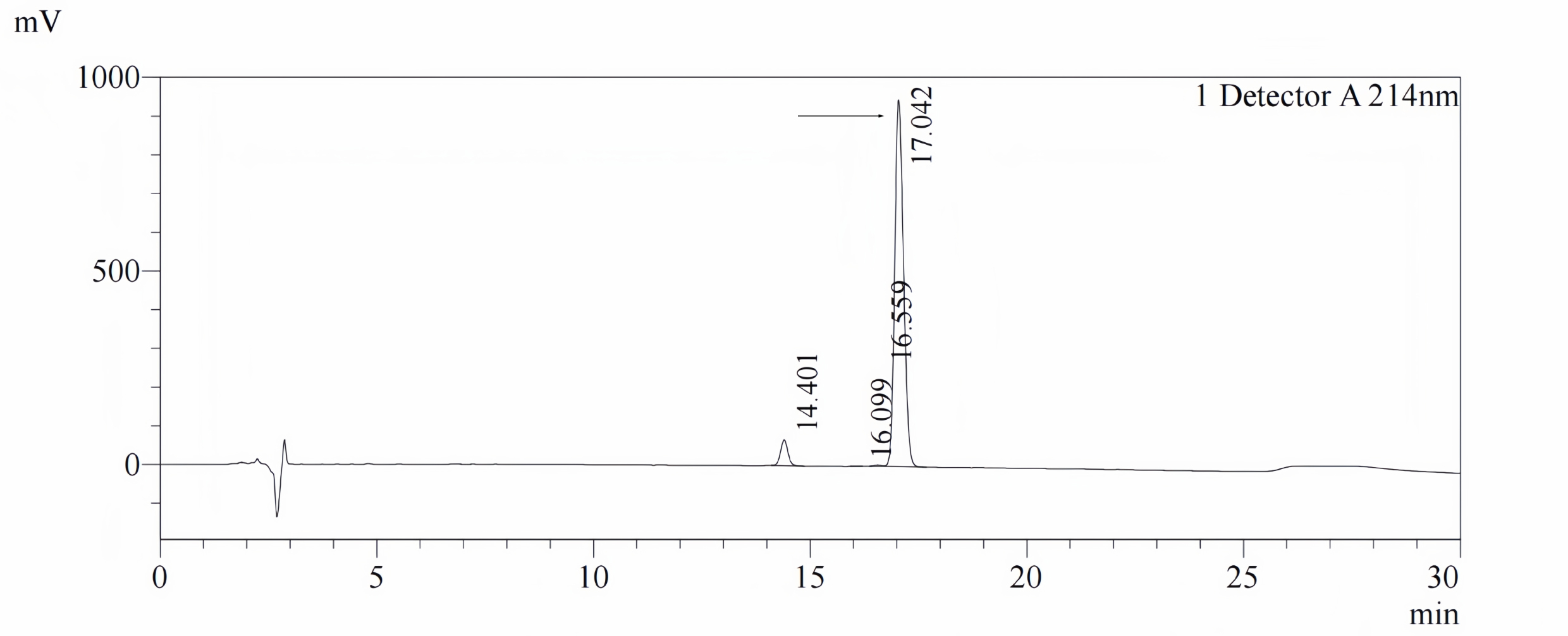

Supplement: Supplementary file 1 [file biomolecules-15-00030-s001.zip › Senegalin-2hplc.jpg]

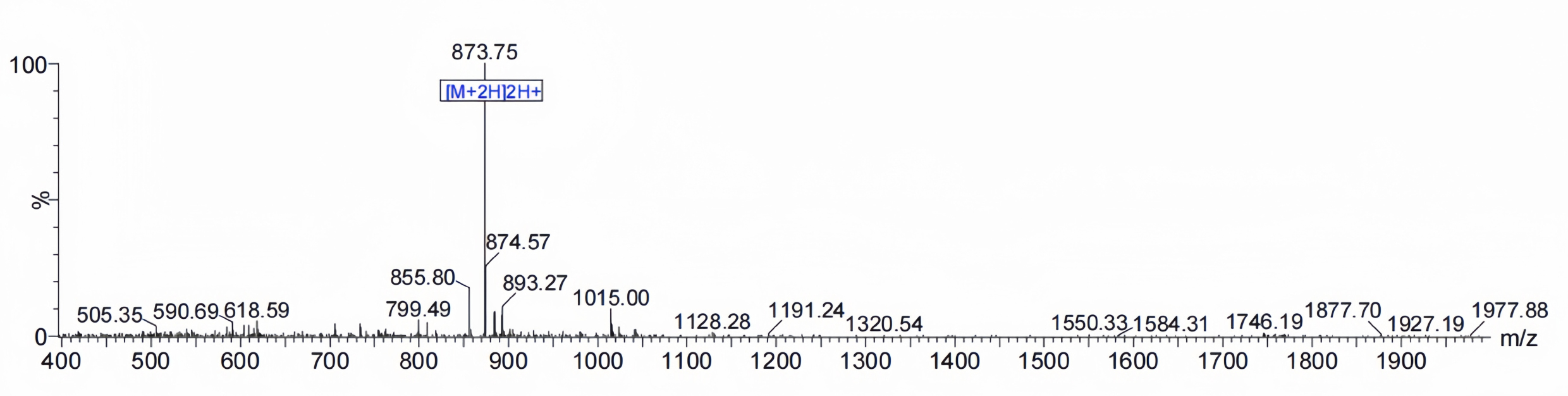

Supplement: Supplementary file 1 [file biomolecules-15-00030-s001.zip › Senegalin-2ms.jpg]
